# Mediterranean Diet Is a Predictor of Progression of Subclinical Atherosclerosis in a Mediterranean Population: The ILERVAS Prospective Cohort Study

**DOI:** 10.3390/nu16213607

**Published:** 2024-10-24

**Authors:** Marina Idalia Rojo-López, Marcelino Bermúdez-López, Eva Castro, Cristina Farràs, Gerard Torres, Reinald Pamplona, Albert Lecube, José Manuel Valdivieso, Elvira Fernández, Josep Julve, Esmeralda Castelblanco, Nuria Alonso, Maria Antentas, Maria Barranco-Altirriba, Alexandre Perera-Lluna, Josep Franch-Nadal, Minerva Granado-Casas, Didac Mauricio, on behalf of the ILERVAS project collaborators

**Affiliations:** 1 Institut de Recerca Sant Pau (IR SANT PAU), Sant Quintí 77-79, 08041 Barcelona, Spain; nut.marina.rojo.l@gmail.com (M.I.R.-L.); jjulve@santpau.cat (J.J.); maantentas@gmail.com (M.A.); 2 Vascular and Renal Translational Research Group, IRBLleida, Renal Research Network (RedInRen. ISCIII), 25198 Lleida, Spain; mbermudez@irblleida.cat (M.B.-L.); ecastro.lleida.ics@gencat.cat (E.C.); valdivielso@irblleida.cat (J.M.V.); edfernandez.lleida.ics@gencat.cat (E.F.); 3 Department of Experimental Medicine, University of Lleida, 25198 Lleida, Spain; reinald.pamplona@udl.cat; 4 Centre d’Atenció Primària Cappont, Gerència Territorial de Lleida, Institut Català de la Salut, 08007 Barcelona, Spain; cfarras.lleida.ics@gencat.cat; 5 Research Support Unit Lleida, Jordi Gol i Gorina Primary Health Care Research Institute Foundation (IDIAPJGol), 08007 Barcelona, Spain; 6 Department of Respiratory Medicine, Arnau de Vilanova University Hospital, 25198 Lleida, Spain; gtorres@gss.cat; 7 Translational Research Group Respiratory Medicine, IRBLleida, University of Lleida, 25198 Lleida, Spain; 8 CIBER of Respiratory Diseases (CIBERES), Instituto de Salud Carlos III (ICSIII), 28029 Madrid, Spain; 9 Department of Endocrinology and Nutrition, Arnau de Vilanova University Hospital, 25198 Lleida, Spain; alecube.lleida.ics@gencat.cat; 10 Obesity and Metabolism Research Group (ODIM), IRBLleida, University of Lleida, 25198 Lleida, Spain; 11 CIBER of Diabetes and Associated Metabolic Diseases (CIBERDEM), Instituto de Salud Carlos III (ISCIII), 08041 Barcelona, Spain; nalonso32416@yahoo.es (N.A.); josep.franch@gmail.com (J.F.-N.); 12 Department of Internal Medicine, Endocrinology, Metabolism and Lipid Research Division, Washington University School of Medicine, St. Louis, MO 63110, USA; esmeraldacas@gmail.com; 13 Department of Endocrinology and Nutrition, Hospital Universitari Germans Trias i Pujol, 08916 Badalona, Spain; 14 Departament of Endocrinology and Nutrition, Hospital de la Santa Creu i Sant Pau, 08041 Barcelona, Spain; barmaria95@gmail.com; 15 Departament d’Enginyeria de Sistemes, Automàtica i Informàtica Industrial, Universitat Politècnica de Catalunya, B2SLab, 08034 Barcelona, Spain; alexandre.perera@upc.edu; 16 Networking Biomedical Research Centre in the Subject Area of Bioengineering, Biomaterials and Nanomedicine, CIBER-BBN, 28029 Madrid, Spain; 17 Institut de Recerca Sant Joan de Déu, Esplugues de Llobregat, 08950 Barcelona, Spain; 18 DAP-Cat Group, Unitat de Suport a la Recerca Barcelona, Fundació Institut Universitari per a la Recerca a l’Atenció Primària de Salut Jordi Gol i Gurina (IDIAPJGol), 08007 Barcelona, Spain; 19 Department of Nursing and Physiotherapy, University of Lleida, 25198 Lleida, Spain; 20 Research Group of Health Care (GreCS), IRBLleida, 25198 Lleida, Spain; 21 Faculty of Medicine, University of Vic-Central University of Catalonia (UVIC-UCC), 08500 Vic, Spain

**Keywords:** lifestyle score, Mediterranean diet, physical activity, atherosclerotic plaque, subclinical atherosclerosis

## Abstract

Atherosclerotic cardiovascular disease remains a major health issue, often developing silently as subclinical atherosclerotic disease (SAD). The Mediterranean diet (MDiet) is known for its cardiovascular benefits, but the combined influence of both MDiet adherence and physical activity (PA) on SAD progression has not been previously documented. **Objective:** We aimed to investigate how adherence to a healthy lifestyle, defined as MDiet adherence and PA level, influences SAD progression in subjects from the ILERVAS cohort follow-up. **Methods:** A study on 3097 participants from the ILERVAS prospective cohort was conducted. MDiet adherence was assessed using the MEDAS score, and PA categories were established using the IPAQ, both categorized into low, moderate, and high levels. Two different lifestyle scores integrating the MDiet and PA categories were built. The presence of atherosclerotic plaques was assessed by carotid and femoral ultrasound examination. Demographic, clinical, and biochemical data were also obtained. Multivariable linear, logistic, and Poisson regression models adjusted for potential confounders were used to analyze the association between the lifestyle scores and SAD progression, as well as the MDiet and PA as separate variables and number of territories with plaque. **Results:** A healthier lifestyle score did not show an effect on SAD progression. However, a higher MEDAS score was associated with a 3% decrease in the number of territories with plaque (IRR 0.97, 95% CI 0.96–0.99, *p* < 0.001), suggesting a protective effect of the adherence to the MDiet. PA did not show a significant association (IRR 1.00, 95% CI 1.00–1.00, *p* = 0.269). Older age, hypertension, dyslipidemia, smoking, and lower eGFR were associated with SAD progression, while the female sex was protective (IRR 0.67, 95% CI 0.63–0.72, *p* < 0.001). **Conclusions:** The findings of this study show that higher adherence to the MDiet is associated with reduced incidence of SAD, indicating its potential role in cardiovascular prevention strategies. Although a higher lifestyle score or physical activity levels did not show any significant effect, promoting the MDiet, alongside managing traditional cardiovascular risk factors, could be an effective public health intervention to prevent atherosclerosis and reduce the burden of cardiovascular disease.

## 1. Introduction

Despite relevant advances in cardiovascular medicine, atherosclerotic cardiovascular disease (CVD) remains the predominant cause of morbidity, mortality, and healthcare expenditure worldwide [1]. Subclinical atherosclerotic disease (SAD) often progresses silently, affecting individuals with low-to-moderate cardiovascular risk at a prevalence of 71.4% [2]. Classical cardiovascular risk factors (CVRFs) such as age, smoking, obesity, diabetes, hypertension, and dyslipidemia contribute to the progression of atherosclerotic lesions through enhancing plaque inflammation and lipid accumulation [3]. The Mediterranean diet (MDiet) is known for its cardiovascular benefits, and several epidemiological studies indicate that higher adherence to this diet is associated with a 9% decrease in overall mortality and CVD mortality [4,5,6,7,8,9]. Despite this, only ~50% of the population in Catalonia adheres to the nutritional recommendations of a typical MDiet [2]. Physical activity (PA) is another relevant modifiable factor for CVD prevention, with regular exercise being associated with lower cardiovascular morbidity and mortality [10]. Despite a relatively high prevalence of healthy PA levels among Catalonians aged 15 to 69, sedentary behavior remains a relevant issue, particularly in older adults, low-income individuals, and those with CVRFs [11,12].

Although the MDiet and PA, assessed independently, are generally thought to have a positive influence on cardiovascular health, conflicting results have been reported in studies regarding the effects on the progression of SAD [13,14,15,16,17,18,19,20,21,22,23]. The MDiet improves endothelial function in individuals with type 2 diabetes (T2D) and prediabetes [13]. However, a sub-analysis of the PREDIMED study found that the MDiet supplemented with nuts or oil showed regression of carotid atherosclerosis only in subjects with greater baseline intima media thickness (IMT) [14]. Additionally, a cross-sectional study detected a higher risk of peripheral artery disease in individuals following the MDiet, though there was bias from including patients who already had the disease [15]. In contrast, higher adherence to the MDiet was linked to a slight reduction in the burden of carotid atherosclerotic plaque (AP) in stroke-free adults [17]. Moreover, a prospective study found that diets high in processed foods increased the risk of SAD, while those rich in vegetables, legumes, and fruits reduced this risk [16]. On the other hand, sedentary behavior and low cardiorespiratory fitness are significant but often overlooked CVRFs [18]. Furthermore, regular PA has been associated with improvement in endothelial function and the progression of AP, along with a reduction in thrombosis [19]. Moreover, participating in moderate PA and avoiding prolonged sedentary behavior has been associated with a reduced risk of carotid plaque formation in randomly selected subjects [20]. Additionally, a greater cardiorespiratory fitness was associated with a higher prevalence of healthy vascular aging and a reduced risk of age-related SAD in healthy men [21]. A longitudinal study found that engaging in vigorous PA, but not light to moderate PA, had a beneficial impact on arterial stiffness in later stages of life [22]. In addition, a meta-analysis of data from subjects with T2D and hypertension found that low-to-moderate PA reduces arterial stiffness, while sedentary behavior increases it [23].

Despite the clear benefits of both the MDiet and PA as individual lifestyle factors [13,14,15,16,17,18,19,20,21,22,23], there is a notable gap in the literature examining their combined effect on SAD progression without the inclusion of other lifestyle factors. To our knowledge, no studies have specifically evaluated the combined effects of MDiet adherence and PA as a composite score in a large prospective cohort of individuals with low-to-moderate cardiovascular risk, with follow-up. These two lifestyle measures are key for the prevention of cardiovascular diseases in the population. The CARDIA study, a 20-year prospective cohort of healthy young adults performed to assess whether lifestyle modifications during early adulthood are linked to the severity and progression of SAD in middle age, showed that a healthier lifestyle (not smoking, regular PA, maintaining a body mass index [BMI] below 25 kg/m^2^, moderate alcohol consumption, and healthy diet) was associated with a reduced risk of coronary artery calcification (CAC) and carotid IMT [24]. Similar findings were observed in a prospective high CVRFs Chinese cohort recruited to investigate the association between a healthy lifestyle and the accelerated progression of carotid plaque, with a median follow-up of 1.2 years [25]. In this study, adherence to healthier lifestyles (non-smoking, regular PA, healthy diet, and an optimal BMI) was associated with a reduced risk of carotid plaque progression [25]. Prospective data from the Study of Women’s Health Across the Nation cohort indicated that adopting a healthy lifestyle (non-smoking, healthy diet, and regular PA) during menopause was associated with reduced SAD [26]. Finally, the Multi-Ethnic Study of Atherosclerosis (MESA), including 2810 subjects without previous CVD, did not find an association of healthier behavior with a progression of arterial stiffness [27].

Given these mixed findings and the absence of studies specifically focused on the combined effect of the MDiet and PA, including a large prospective cohort, further research is needed to explore how these lifestyle factors influence the progression of atherosclerosis. We previously evaluated the relationship between the MDiet and the presence of SAD in a Mediterranean population with low-to-moderate CVRFs in a cross-sectional study [28]. In the present study, we hypothesized that a healthy lifestyle characterized by higher adherence to the MDiet and PA is associated with a lower risk of progression of SAD. Hence, we aimed to evaluate the association between a healthier lifestyle and the progression of SAD in the ILERVAS cohort at 4-year follow-up.

## 2. Materials and Methods

### 2.1. Study Design

The prospective ILERVAS study (ClinicalTrials.gov Identifier: NCT03228459) examined the progression of SAD, with a focus on evaluating its prevalence and prognosis. Out of an initial cohort of 8330 participants enrolled in primary healthcare centers in Lleida, Spain, between January 2015 and December 2018, a final cohort of 8116 individuals with available data on MDiet adherence, PA, and AP at baseline was selected [2,29] (Supplementary Figure S1). For the present analysis, comprehensive data on MDiet adherence, PA, and AP were available for 3097 subjects during the 4-year follow-up period.

To be included, participants had to be aged 45 to 70 years with at least one CVRF. These risk factors included obesity, dyslipidemia (determined by the use of lipid-lowering medication), or a disorder related to lipoprotein metabolism as defined by the International Classification of Diseases codes [30]; other risk factors considered were hypertension (defined as systolic blood pressure [sBP] ≥ 140 mmHg and/or diastolic blood pressure [dBP] ≥ 90 mmHg), smoking habits (including current and former smokers, with former smokers defined as those who had quit smoking at least one year prior to recruitment) [2], or having a first-degree relative with premature CVD. Participants were excluded if they had any type of diabetes, chronic kidney disease, active neoplasia, a life expectancy of less than 18 months, or were pregnant. Additionally, individuals with a history of previous CVD, including angina, myocardial infarction, stroke, peripheral artery disease, heart failure, or any vascular surgery/procedure, were also excluded [2,29]. The prescribed treatments for hypertension, lipid reduction, and antithrombotic therapy were obtained from prescription and pharmacy invoicing records supplied by the Catalan Health Service, which are annually integrated into the Information System for Research in Primary Care (SIDIAP) database. Antihypertensive medications included ACE inhibitors, diuretics, ARBs, beta-blockers, calcium channel blockers, and other antihypertensives drugs. Lipid-lowering treatments comprised statins, fibrates, ezetimibe, and omega-3 fatty acids. Antithrombotic therapy involved the use of anticoagulants or antiplatelet agents [30].

The ILERVAS protocol was approved by the Ethics committee of University Hospital Arnau de Vilanova (First visit: CEIC-1410, 19/12/2014; Follow-up: CEIC-2015, 20/12/2018). Written informed consent was obtained from all participants. This study adhered to the ethical principles outlined in the Declaration of Helsinki and complied with Spain’s data protection legislation.

### 2.2. Assessment of the Mediterranean Diet Adherence

Adherence to the MDiet was assessed using the validated 14-item Mediterranean Diet Adherence Screener (MEDAS) questionnaire, recognized as a reliable tool developed during the PREDIMED trial [31]. Validation of the MEDAS questionnaire included comparing it with the validated Food Frequency Questionnaire (FFQ) in the PREDIMED study; the findings showed a strong correlation between the MEDAS score and the PREDIMED FFQ score, indicating the accuracy of the questionnaire in assessing dietary patterns [31]. The questionnaire includes two questions regarding eating behaviors unique to the Spanish MDiet and 12 questions about the frequency of food consumption [31]. This tool assesses how often participants consume olive oil, nuts, fruits, vegetables, sofrito (a tomato sauce cooked with virgin olive oil, onions, garlic, and spices), fish, legumes, and wine [31,32]. Moreover, the questionnaire considers the weekly intake of meat, meat products, animal fat, commercial pastries, and sugar-sweetened beverages as part of the composite score [31,32]. Adherence to the MDiet was classified into three categories based on the MEDAS score as low (0–6 points), moderate (from 7 to 9 points), and high (≥10 points) [31,33].

### 2.3. Physical Activity

PA was assessed using the abbreviated version of the International Physical Activity Questionnaire (IPAQ), a validated tool for gauging PA levels among adults in Spain [34]. The Metabolic Equivalent Tasks (METS) were derived from the IPAQ data by multiplying the minutes spent on each activity over the course of 7 days [34]. Physical activity was classified into three categories: low, moderate, and high. This classification was determined by multiplying the MET intensity (3.3 METs for walking [low], 4.0 METs for moderate-intensity activities [moderate], and 8.0 METs for vigorous-intensity activities [high]) by the duration in minutes of each activity performed over a seven-day period [34,35].

### 2.4. Clinical Variables

Trained researchers obtained anthropometric measurements following standardized procedures. Body weight and height were measured with participants wearing light clothing and no shoes. Waist circumference was evaluated while participants stood, measured horizontally between the lower rib and the iliac crest. Based on the American Heart Association guidelines, waist circumference was categorized as either a low or high cardiovascular risk: high-risk waist was defined as ≥102 cm for men (increased risk) and ≥88 cm for women (increased risk) [36]. Standard tools were utilized, offering a precision of 0.5 kg for the body scale, 1.0 cm for the stadiometer, and 0.1 cm for the non-elastic measuring tape [31,32]. The BMI was calculated, with obesity defined as a BMI of ≥30 kg/m^2^ [37]. Blood pressure was measured using an automated device [Omron M6 Comfort HEM-7221-E (Omron Healthcare, Kyoto, Japan)] three times with a 2 min gap between each reading, and the average of the last two readings was recorded. Hypertension was defined as having a reading of ≥140/90 mmHg [29]. Blood samples were obtained and clinical parameters were examined using standardized protocols, as outlined in a prior publication [29]. Cardiovascular risk was evaluated using the REGICOR score, a calibrated version of the Framingham prediction model, specifically designed to predict coronary events over a 5-year period in the Spanish population [38]. To estimate the risk, the algorithm considers diabetes status, gender, age, systolic and diastolic blood pressure, total cholesterol, and HDL cholesterol levels [38].

### 2.5. Subclinical Atherosclerotic Disease

The Doppler Ultrasound Vivid-I (General Electric Healthcare, Waukesha, WI, USA), fitted with a 12L RS/4–13 MHz linear probe, was utilized by qualified sonographers for ultrasound imaging of vascular territories in the carotid arteries (common carotid, bifurcation or bulb, internal carotid, and external carotid) as well as in the femoral arteries (common and superficial). Additionally, it included a module for measuring intima media thickness [39]. AP was defined as focal intima-media intrusion into the lumen measuring ≥1.5 mm [39]. SAD was defined by the detection of plaque in any of the twelve territories [40]. The number of plaques was classified as follows: no plaque was indicated when no visible plaque was observed in the sample or area being evaluated; one plaque was indicated by the presence of a single visible plaque in the sample or area being evaluated; and multiple plaques were indicated when more than one visible plaque was observed in the sample or area being evaluated. The AP condition was assessed by examining its progression based on changes in the presence of AP across one or more territories during the follow-up period. The progression of SAD was determined by an increase in the number of vascular territories with detected AP. Participants were classified into two groups: those who demonstrated progression and those who did not.

### 2.6. Lifestyle Score

The lifestyle score was constructed by combining quartiles of the MEDAS and PA measured in METS. According to the PREDIMED study, the classification of MEDAS to determine the adherence to the MDiet was scored with 0 points for the low adherence group (from 0 to 6 points), 1 point for the moderate group (from 7 to 9 points), and 2 points for the high group (≥10 points) [41]. PA was stratified into quartiles, representing different levels of physical activity, with each quartile assigned 1 to 4 points (1 for the lowest quartile and 4 for the highest). The use of quartiles allowed us to capture the relative distribution of PA within the cohort and facilitate comparisons between individuals with different activity levels and MDiet categories. The lifestyle score was calculated by combining all the MDiet groups with all PA quartiles, resulting in a score from 1 to 6 points. Finally, the lifestyle score was classified into four groups as follows: unhealthy (1 and 2 points), mildly healthy lifestyle (3 points), moderately healthy (4 points), and highly healthy (5 and 6 points). Additionally, we calculated a composite lifestyle score (summarized lifestyle score) by standardizing MEDAS and METS data into z-scores. These z-scores were then averaged to create a single metric, reflecting overall lifestyle behavior. Higher scores indicate healthier diet and greater PA, while lower scores suggest poorer lifestyle habits.

### 2.7. Statistical Analysis

The Shapiro–Wilk test was employed to assess the normality of continuous variables. Continuous variables with a normal distribution are reported as mean and standard deviation, while categorical variables are presented as absolute frequencies. Group differences were evaluated using the chi-square test or Fisher’s test for categorical variables, and the T-Student or ANOVA test were used for continuous variables. Additionally, the Levene test was used to examine the equality of variances. In the next step, bivariable models were utilized to examine the unadjusted association between variables and the outcome, which is the progression of SAD. Following this, adjusted multivariable logistic models were performed to assess the relationship between lifestyle at baseline of this study and the progression of SAD at follow-up. Additionally, an adjusted multivariable Poisson analysis was conducted to investigate the association between either the categorical lifestyle or MEDAS score and PA in METS at baseline, and the burden of SAD in terms of the number of territories affected by AP. The multivariable models were adjusted for clinical confounding factors, including age, gender, hypertension, dyslipidemia, tobacco use, waist circumference by gender, glycemic control, and estimated glomerular filtration rate (eGFR). The odds ratio (OR) and 95% confidence intervals (CIs) are shown in bivariable and logistic models. The incidence rate ratio (IRR) and corresponding 95% CI are presented in the Poisson models. All statistical analyses were conducted using STATA v.16 software and R Statistical Software (v. 4.1.2), with a significance threshold set at 0.05 [42,43].

## 3. Results

The clinical characteristics of the study groups are shown in Table 1. The group of subjects with an unhealthy lifestyle were younger (mean age 56.8 years, *p* = 0.005) and had fewer women (46.4%, *p* < 0.001). This group had a higher prevalence of obesity (32.5%, *p* = 0.009), higher sBP (mean 131.2 mmHg, *p* = 0.048) and dBP (mean 82.4 mmHg, *p* < 0.001), and a higher prevalence of tobacco exposure (62.3%, *p* < 0.001). They showed lower adherence to the MDiet (mean MEDAS score 6.2, *p* < 0.001) and lower PA levels (3% moderate activity, nonhigh activity, *p* < 0.001). Their kidney function was higher (eGFR 92.0 mL/min/1.73 m^2^, *p* < 0.001) in comparison with the other groups. Overall, as the healthiness of lifestyle increased from the unhealthy to healthier lifestyle groups, there were trends for an older age, a higher proportion of women, lower prevalence of obesity and smoking, higher adherence to the MDiet, and higher levels of PA.

In the bivariable analysis, several factors emerged as significant predictors of the progression of SAD (Table 2). Older age was strongly associated with an increased likelihood of SAD progression (OR 1.03, 95% CI 1.01–1.04, *p* < 0.001). Additionally, a higher REGICOR score (OR 1.18, 95% CI 1.13–1.22, *p* < 0.001), hypertension (OR 1.23, 95% CI 1.05–1.43, *p* = 0.009), and sBP (OR 1.01, 95% CI 1.01–1.02, *p* < 0.001) and dBP (OR 1.02, 95% CI 1.01–1.02, *p* < 0.001) were significantly associated with an increased risk of the progression of SAD. Furthermore, dyslipidemia (OR 1.29, 95% CI 1.11–1.43, *p* = 0.001) and smoking (OR 1.30, 95% CI 1.06–1.43, *p* = 0.007) also showed significant associations with progression. Moreover, higher glycemia (OR 1.67, 95% CI 1.36–2.05, *p* < 0.001), total cholesterol (TC) (OR 1.24, 95% CI 1.13–1.37, *p* < 0.001), LDL cholesterol (OR 1.25, 95% CI 1.11–1.42, *p* < 0.001), and triglycerides (OR 1.15, 95% CI 1.04–1.27, *p* = 0.005) were also significant risk factors for SAD progression. Conversely, the female sex was associated with a protective effect (OR 0.80, 95% CI 0.69–0.93, *p* = 0.003).

In the logistic multivariable model for the progression of SAD (Table 3), none of the lifestyle categories showed a statistical association. However, older age was strongly associated with a higher risk of SAD progression (OR 1.04, 95% CI 1.02–1.05, *p* < 0.001), as was male gender (OR 1.26, 95% CI 1.06–1.49, *p* = 0.007). Additionally, hypertension (OR 1.23, 95% CI 1.05–1.43, *p* = 0.007), dyslipidemia (OR 1.35, 95% CI 1.16–1.60, *p* < 0.001), and smoking (OR 1.37, 95% CI 1.16–1.61, *p* < 0.001) were also associated with a higher risk of SAD progression.

The logistic model for SAD progression with the MDiet and PA, separately (Supplementary Table S1), showed no significant association between the MEDAS score (OR 0.98, 95% CI 0.93–1.02, *p* = 0.313) or PA (METS) (OR 1.00, 95% CI 1.00–1.00, *p* = 0.342) and the progression of SAD. However, age (OR 1.04, 95% CI 1.02–1.05, *p* < 0.001), hypertension (OR 1.26, 95% CI 1.06–1.49, *p* = 0.007), dyslipidemia (OR 1.35, 95% CI 1.15–1.60, *p* < 0.001), and smoking (OR 1.37, 95% CI 1.16–1.62, *p* < 0.001) were all significantly associated with plaque progression. Additionally, being of female sex (OR 0.79, 95% CI 0.66–0.94, *p* = 0.009) was a protective factor against plaque progression.

In the linear regression analysis of the difference in the number of territories between baseline and the follow-up, the summarized lifestyle score did not show a significant association (*p* = 0.42) (Supplementary Table S2). However, age (*p* < 0.001), smoking (*p* < 0.001), hypertension (*p* = 0.001), dyslipidemia (*p* = 0.01), and higher HbA1c (*p* < 0.001) were positively associated with an increase in the number of plaque territories. Conversely, female sex and waist circumference were associated with a decreased number of plaque territories (*p* < 0.001 and *p* = 0.01, respectively). Finally, eGFR did not show any significant association (*p* = 0.42).

**Figure 1:**
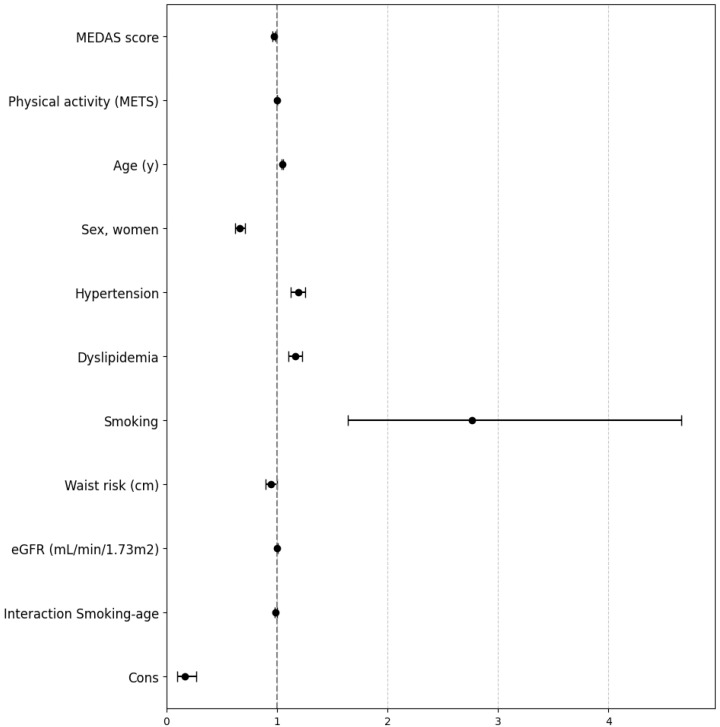
Forest plot presents the results of the multivariable Poisson model for the analysis of the factors associated with the number of atherosclerotic plaques at the follow-up including baseline MEDAS score and physical activity. eGFR, estimated glomerular filtration rate; IRR, incidence rate ratio; 95% CI, confidence interval. Cons estimates baseline incidence rate.

Additionally, a Poisson regression analysis was conducted to explore the interactions between age and smoking as potential confounding factors. The findings indicated that a higher MEDAS score was associated with a 3% reduction in the IRR (IRR 0.97, 95% CI 0.96–0.99, *p* < 0.001). Similarly, older age (IRR 1.05, 95% CI 1.04–1.06, *p* < 0.001), hypertension (IRR 1.19, 95% CI 1.13–1.26, *p* < 0.001), dyslipidemia (IRR 1.16, 95% CI 1.10–1.22, *p* < 0.001), smoking (IRR 2.77, 95% CI 1.64–4.66, *p* < 0.001), and lower eGFR (IRR 1.00, 95% CI 1.00–1.00, *p* = 0.038) were all associated with a greater number of territories affected by AP at the follow-up visit. Conversely, female sex (IRR 0.67, 95% CI 0.63–0.72, *p* < 0.001) was associated with a protective effect against plaque progression. Further, the interaction between smoking and age was marginally significant (IRR 0.99, 95% CI 0.98–1.00, *p* = 0.014). On the other hand, PA did not show any relationship with the number of territories with AP at the follow-up (IRR 1.00, 95% CI 1.00–1.00, *p* = 0.218). The study population exhibited a lower baseline prevalence rate (IRR 0.16, 95% CI 0.10–0.27, *p* < 0.001) after adjusting for all other factors in the model (Figure 1, Supplementary Table S3).

The multivariable Poisson model for the analysis between lifestyle at baseline and the number of territories with AP at the follow-up revealed no significant association between lifestyle categories (unhealthy, mild, moderate, or healthy) and the number of territories with AP at the follow-up (Supplementary Table S4). However, age (IRR 1.04, 95% CI 1.04–1.05, *p* < 0.001), female sex (IRR 0.67, 95% CI 0.63–0.71, *p* < 0.001), hypertension (IRR 1.19, 95% CI 1.12–1.25, *p* < 0.001), dyslipidemia (IRR 1.16, 95% CI 1.10–1.22, *p* < 0.001), and smoking (IRR 1.45, 95% CI 1.36–1.53, *p* < 0.001) were all significantly associated with the number of territories with AP. Additionally, eGFR had a minor but significant effect (IRR 1.00, 95% CI 1.00–1.00, *p* = 0.040).

## 4. Discussion

Our study did not reveal an association between a healthier lifestyle, measured by adherence to the Mediterranean diet and physical activity, and the progression of SAD in any of the composite scores. However, a higher MEDAS score at baseline was associated with a decreased IRR of the number of territories with AP at the follow-up, while PA measured by METS showed no significant association. Additionally, older age, hypertension, dyslipidemia, smoking, and a lower eGFR were associated with a higher risk of SAD progression, whereas female gender showed a protective effect. The sensitivity analyses of the interactions between the MDiet, PA, and confounding factors like age and smoking showed a marginal association with atherosclerosis risk.

Contrary to our findings, healthier lifestyle scores that included variables such as non-smoking, regular PA, healthy diet, optimal BMI, and even moderate alcohol consumption have been associated with a reduced risk of SAD [24,25,26]. However, similar to our study, the MESA study showed that having a better score in healthy behaviors may not necessarily be linked to a reduction in the progression of SAD [27]. It is important to note that our score only included PA and diet, and considering additional factors might impact the score’s ability to predict SAD progression. However, diet and physical exercise are fundamental for measuring healthy lifestyles. Unlike other lifestyle scores that include alcohol and smoking alongside diet and exercise, our score integrates alcohol consumption within the MEDAS score. Regarding smoking, our models account for this variable, following common practice in other studies. Consequently, we have chosen to focus our score exclusively on these two key aspects of lifestyle.

The results of our study emphasize the importance of adherence to a MDiet compared to the overall lifestyle score in the progression of SAD, which is in line with previous research [13,14,15,17,44,45,46,47]. Notably, an intervention involving a MDiet supplemented with nuts was associated with either the reversal or slower progression of carotid IMT and AP [44]. Additionally, participants in the prospective PESA study who adhered to a MDiet showed a 1.3-fold lower prevalence of SAD compared with those following the social-business eating pattern [45]. Similarly, the cross-sectional Aragon Workers’ Health Study showed that the adherence to the MDiet had a protective association against SAD in men, independent of other risk factors [46]. Furthermore, it has also been reported that, in the long term, adherence to the MDiet lowers inflammatory biomarkers linked to the development of SAD in high-risk elderly individuals, according to an intervention study [47].

According to the IPAQ guidelines, a high PA category is defined as achieving a total of at least 3000 METS per week [48]; in our study, the healthy lifestyle group achieved around 2940 METS. Consistent with this, in another study, physically active individuals who achieved more than 2000 MET minutes per week (i.e., very high PA levels) exhibited a higher prevalence of SAD [49]. Similarly, earlier research has demonstrated a higher frequency of SAD in endurance athletes when compared with control groups [49,50,51]. Interestingly, two randomized controlled trials have examined the effects of more intensive exercise on plaque regression, showing plaque reduction in both trials, although neither study found a significant difference between the treatment groups [50,51]. However, a cross-sectional study suggested that while athletic men show higher CAC scores and greater coronary plaques, these may indicate both potential harmful and protective benefits against plaque rupture and heart attacks [52]. Furthermore, exercise intensity, rather than amount of exercise, has been associated with coronary atherosclerosis progression [53]. Interestingly, very vigorous exercise was associated with an increased CAC and calcified plaque progression, while vigorous exercise was associated with decreased CAC progression [53]. Although in our study PA measured by METS did not show an association with plaque progression, it is important to consider that other aspects of PA, such as intensity and frequency, might still play crucial roles in cardiovascular health [54]. It is possible that our score did not accurately capture the effect of a healthy lifestyle on the progression of AP because there are significantly more subjects with moderate-to-high adherence to a MDiet compared with those with moderate-to-high PA. However, the data from our study showing that the MDiet has a significant effect on SAD progression (while PA did not) suggest that maintaining a healthy diet is important for cardiovascular health just as much (if not more) than engaging in regular (moderate) PA.

Clinical factors associated with SAD progression were examined in detail. Age emerged as a strong predictor of SAD progression in the current study. This aligns with established knowledge that cardiovascular risk increases with age [55,56,57]. Hypertension, dyslipidemia, and smoking were also significant predictors, emphasizing the need for effective management of these conditions to mitigate SAD progression. Interestingly, the female sex was consistently associated with a lower risk of disease progression, highlighting potential gender differences in CVD risk profiles. These findings are consistent with previous studies indicating that plaque size and burden are typically greater in men, especially at a younger age, with women tending to reach comparable levels later in life [58]. In addition, the prevalence of SAD has been described to be 1.9 times higher in men compared with women and increases significantly with age in both genders [59].

The current study has several limitations. The ILERVAS population consisted of middle-aged individuals with low-to-moderate CVD risk, potentially limiting the generalizability of our findings to a broader population. Additionally, the MEDAS questionnaire and IPAQ were completed by the participants, which could introduce errors that may affect the accuracy of our data. Another significant limitation was the lack of detailed metrics on PA, particularly regarding intensity and frequency. This information could have better informed a potential association between PA and AP progression in our study. Nevertheless, our study has several strengths. The entire ILERVAS study cohort comprised a large population with follow-up data, randomly selected using a stratified sampling approach based on primary care records to minimize selection bias. Furthermore, we conducted an analysis on the number of vascular territories affected by AP, which is considered a valid and robust noninvasive tool for predicting CVD. Moreover, the MDiet serves as the traditional eating pattern in this region, ensuring that our data were not influenced by prior clinical interventions. Additionally, the MEDAS score is a validated tool to assess adherence to the MDiet that is quick and easy for participants to complete.

## 5. Conclusions

In conclusion, our study provides valuable insights into the progression of SAD, emphasizing the relevant impact of lifestyle factors. Adherence to the MDiet emerged as a critical factor influencing disease progression, whereas higher levels of PA did not exhibit an association with plaque progression, indicating that the relationship between PA and disease advancement may be more complex than previously understood. Age, hypertension, dyslipidemia, smoking, and lower kidney function were consistently identified as strong predictors of the progression of SAD, highlighting the importance of managing these risk factors. Moreover, our findings consistently demonstrated a protective effect of the female sex against disease progression. Overall, this study underscores the need for a holistic approach to prevent the progression of SAD. It also highlights the importance of integrating lifestyle modifications and risk factor management, with a particular emphasis on personalized interventions that prioritize dietary changes, specifically those based on the MDiet.

## Figures and Tables

**Table 1:** Clinical characteristics of participants according to their lifestyle at baseline.

Characteristics	Unhealthy Lifestyle N = 1051	Mildly Healthy Lifestyle N = 785	Moderately Healthy Lifestyle N = 783	Highly Healthy Lifestyle N = 478	*p*
Age (years)	56.8 (6.2)	57.6 (6.5)	57.7 (6.2)	57.7 (6.0)	0.005
Sex (women)	488 (46.4)	411 (52.4)	446 (57)	260 (54.4)	<0.001
Caucasian	1046 (99.5)	783 (99.8)	782 (99.9)	478 (100)	0.298
BMI (kg/m^2^)	29.5 (5.1)	28.7 (4.8)	28.7 (4.8)	28.8 (4.7)	0.292
Waist (cm)	101.9 (12.0)	100.0 (11.6)	99.6 (11.3)	99.5 (11.2)	0.196
Obesity	341 (32.5)	199 (25.4)	232 (28.5)	147 (30.8)	0.009
Hypertension	402 (38.3)	311 (39.6)	304 (38.8)	190 (39.8)	0.920
sBP (mmHg)	131.2 (15.5)	131.1 (16.9)	130.2 (16.7)	129.8 (16.0)	0.048
dBP (mmHg)	82.4 (9.2)	81.3 (9.4)	81.0 (8.9)	80.3 (9.1)	<0.001
Smoking	655 (62.3)	445 (56.7)	414 (52.9)	240 (50.2)	<0.001
Glucose (mmol/L)	5.4 (1.0)	5.4 (0.9)	5.3 (1.0)	5.3 (0.8)	0.504
Dyslipidemia	535 (50.9)	447 (56.9)	429 (54.8)	272 (56.9)	0.037
TC (mmol/L)	5.8 (1.1)	5.8 (1.1)	5.7 (1.0)	5.7 (1.1)	0.754
HDL (mmol/L)	1.7 (0.5)	1.8 (0.5)	1.8 (0.5)	1.7 (0.5)	0.203
LDL (mmol/L)	3.2 (0.8)	3.2 (0.9)	3.2 (0.8)	3.2 (0.9)	0.936
TG (mmol/L)	1.8 (1.2)	1.8 (1.2)	1.7 (0.9)	1.8 (1.4)	0.160
AST	18.2 (7.9)	19.5 (12.4)	18.3 (7.8)	18.5 (6.8)	0.135
ALT	11.2 (6.4)	11.6 (11.5)	10.9 (7.0)	10.3 (5.2)	0.293
eGFR (mL/min/1.73 m^2^)	92.0 (14.2)	90.0 (14.0)	90.0 (14.1)	89 (14.1)	<0.001
REGICOR score	4.4 (2.1)	4.3 (2.1)	4.3 (2.2)	4.3 (2.0)	0.512
MEDAS score	6.2 (1.4)	7.0 (1.6)	7.2 (1.6)	8.5 (1.3)	<0.001
MedDiet Adherence					<0.001
Low	663 (63.1)	286 (36.4)	236 (30.1)	0 (0.0)	
Moderate	388 (36.9)	457 (58.2)	490 (62.6)	363 (75.9)	
High	0 (0.0)	42 (5.4)	57 (7.3)	115 (24.1)	
IPAQ (METS)	105.0 (183.7)	586.5 (389.5)	1648.9 (1646.5)	2940.5 (2204.3)	<0.001
Physical activity					<0.001
Low	1020 (97.1)	529 (67.4)	213 (27.2)	48 (10.0)	
Moderate	31 (3.0)	256 (32.6)	479 (61.2)	275 (57.5)	
High	0 (0.0)	0 (0.0)	91 (11.6)	155 (32.4)	
Total plaques	1.9 (2.0)	1.8 (1.9)	1.7 (1.9)	1.7 (1.9)	0.217
Number of plaques					0.210
No plaque	315 (30)	268 (34.1)	252 (32.2)	165 (34.5)	
One plaque	225 (21.4)	141 (18)	171 (21.8)	100 (20.9)	
Multiple plaques	511 (48.6)	376 (47.9)	360 (46.0)	213 (44.6)	
Plaque progression	711 (67.7)	505 (64.3)	519 (66.3)	316 (66.1)	0.529

Data are shown as n (%) for categorical variables and mean (SD) for continuous variables. ALT, alanine aminotransferase; AST, aspartate aminotransferase; BMI, body mass index; dBP, diastolic blood pressure; eGFR, estimated glomerular filtration rate; HDL, high density lipoprotein; LDL, low density lipoprotein; *p*, *p*-value; REGICOR, cardiovascular risk score; sBP, systolic blood pressure; TC, total cholesterol; TG, triglycerides. Smoking habits include current and former smokers.

**Table 2:** Bivariable analysis between variables at baseline and the progression of plaque at the follow-up.

Variables	Progression	
	OR (95% CI)	*p*
Age (years)	1.03 (1.01–1.04)	<0.001
Sex (women)	0.80 (0.69–0.93)	0.003
Caucasian	0.50 (0.13–2.04)	0.340
Hypertension	1.23 (1.05–1.43)	0.009
Dyslipidemia	1.29 (1.11–1.43)	0.001
Smoking	1.30 (1.06–1.43)	0.007
Physical activity (METS)	1.00 (1.00–1.00)	0.480
BMI (kg/m^2^)	0.99 (0.97–1.00)	0.173
Waist (cm)	1.00 (0.99–1.00)	0.535
sBP (mmHg)	1.01 (1.01–1.02)	<0.001
dBP (mmHg)	1.02 (1.01–1.02)	<0.001
HbA1c (%)	1.67 (1.36–2.05)	<0.001
TC (mmol/L)	1.24 (1.13–1.37)	<0.001
HDL (mmol/L)	1.03 (0.83–1.27)	0.804
LDL (mmol/L)	1.25 (1.11–1.42)	<0.001
TG (mmol/L)	1.15 (1.04–1.27)	0.005
eGFR (mL/min/1.73 m^2^)	1.00 (1.00–1.00)	0.098
REGICOR score	1.18 (1.13–1.22)	<0.001
MEDAS score	0.98 (0.94, 1.03)	0.430
Lifestyle		
Mild lifestyle	0.86 (0.71–1.05)	0.137
Moderate lifestyle	0.94 (0.77–1.14)	0.538
Healthy lifestyle	0.93 (0.74–1.17)	0.552

Unhealthy lifestyle was the reference group. BMI, body mass index; dBP, diastolic blood pressure; eGFR, estimated glomerular filtration rate; HbA1c, glycated hemoglobin; HDL, high density lipoprotein; IPAQ, international physical activity questionnaire; LDL, low density lipoprotein; METS, Metabolic Equivalent Task; *p*, *p*-value; sBP, systolic blood pressure; TC, total cholesterol; TG, triglycerides; OR, odds ratio; CI, confidence interval.

**Table 3:** Logistic model for the analysis between lifestyle at baseline and progression of plaque at follow-up.

Variables	Progression	
	OR (95% CI)	*p*
Lifestyle		
Mildly healthy lifestyle	0.83 (0.68–1.01)	0.068
Moderately healthy lifestyle	0.94 (0.78–1.15)	0.549
Highly healthy lifestyle	0.91 (0.72–1.16)	0.477
Age (years)	1.04 (1.02–1.05)	<0.001
Sex (women)	0.79 (0.66–0.94)	0.007
Hypertension	1.26 (1.06–1.49)	0.007
Dyslipidemia	1.35 (1.16–1.60)	<0.001
Smoking	1.37 (1.16–1.61)	<0.001
High risk waist (cm)	0.83 (0.70–0.99)	0.034
eGFR (mL/min/1.73 m^2^)	1.00 (1.00–1.00)	0.605
Cons	0.27 (0.09–0.79)	0.017

Unhealthy lifestyle was the reference group. CI, confidence interval; eGFR, estimated glomerular filtration rate; OR, odds ratio; *p*, *p*-value. Cons estimates baseline incidence rate. Note: This logistic regression model assesses the association between different lifestyle categories at baseline (mildly, moderately, and highly healthy) and the progression of AP at the follow-up.

## Data Availability

The data presented in this study are available on request from the corresponding author.

## Supplementary Materials

The following supporting information can be downloaded at: https://www.mdpi.com/article/10.3390/nu16213607/s1, Figure S1: Flow chart of the study population; Table S1: Multivariable logistic model for the analysis between the Mediterranean diet and physical activity at baseline and progression of plaque; Table S2: Model of linear regression analysis of the number of plaque territories and the summarized score between the baseline and the follow-up; Table S3: Multivariable Poisson model for the analysis between the Mediterranean diet, physical activity and the interaction of smoking and age at baseline and number of territories with plaque at follow-up; Table S4: Multivariable Poisson model for the analysis between Lifestyle at baseline and number of territories with plaque at follow-up.

## References

[1] Mendieta G., Pocock S., Mass V., Moreno A., Owen R., García-Lunar I., López-Melgar B., Fuster J.J., Andres V., Pérez-Herreras C. (2023). Determinants of Progression and Regression of Subclinical Atherosclerosis over 6 Years. J. Am. Coll. Cardiol..

[2] Bermúdez-López M., Martínez-Alonso M., Castro-Boqué E., Betriu À., Cambray S., Farràs C., Barbé F., Pamplona R., Lecube A., Mauricio D. (2020). Subclinical Atheromatosis Localization and Burden in a Low-to-Moderate Cardiovascular Risk Population: The ILERVAS Study. Rev. Esp. Cardiol..

[3] Chait A., den Hartigh L.J. (2020). Adipose Tissue Distribution, Inflammation and Its Metabolic Consequences, Including Diabetes and Cardiovascular Disease. Front. Cardiovasc. Med..

[4] Lairon D. (2007). Intervention Studies on Mediterranean Diet and Cardiovascular Risk. Mol. Nutr. Food Res..

[5] Vincent-Baudry S., Defoort C., Gerber M., Bernard M.-C., Verger P., Helal O., Portugal H., Planells R., Grolier P., Amiot-Carlin M.-J. (2005). The Medi-RIVAGE Study: Reduction of Cardiovascular Disease Risk Factors after a 3-Mo Intervention with a Mediterranean-Type Diet or a Low-Fat Diet. Am. J. Clin. Nutr..

[6] Estruch R., Ros E., Salas-Salvadó J., Covas M.-I., Corella D., Arós F., Gómez-Gracia E., Ruiz-Gutiérrez V., Fiol M., Lapetra J. (2013). Primary Prevention of Cardiovascular Disease with a Mediterranean Diet. N. Engl. J. Med..

[7] Pérez-Jiménez F., López-Miranda J., Pinillos M.D., Paz-Rojas E., Montilla P., Marín C., Velasco M.J., Blanco-Molina A., Jiménez Perepérez J.A., Gómez P. (2001). A Mediterranean and a High-Carbohydrate Diet Improve Glucose Metabolism in Healthy Young Persons. Diabetologia.

[8] Esposito K., Maiorino M.I., Bellastella G., Chiodini P., Panagiotakos D., Giugliano D. (2015). A Journey into a Mediterranean Diet and Type 2 Diabetes: A Systematic Review with Meta-Analyses. BMJ Open.

[9] Sofi F., Cesari F., Abbate R., Gensini G.F., Casini A. (2008). Adherence to Mediterranean Diet and Health Status: Meta-Analysis. BMJ.

[10] Narendrula A., Brinza E., Horvat Davey C., Longenecker C.T., Webel A.R. (2024). Relationship between Objectively Measured Physical Activity and Subclinical Cardiovascular Disease: A Systematic Review. BMJ Open Sport. Exerc. Med..

[11] Gonzalez-Viana A., Violan Fors M., Castell Abat C., Rubinat Masot M., Oliveras L., Garcia-Gil J., Plasencia A., Cabezas Peña C. (2018). Promoting Physical Activity through Primary Health Care: The Case of Catalonia. BMC Public Health.

[12] (2023). Departament de Salut L’estat de Salut, Els Comportaments Relacionats Amb La Salut i l’ús de Serveis Sanitaris a Catalunya, 2022 Resum Executiu Dels Principals Resultats de l’ESCA Del 2022 Direcció General de Planificació i Recerca En Salut; Barcelona. https://scientiasalut.gencat.cat/bitstream/handle/11351/11405/enquesta_salut_catalunya_resum_executiu_ca_2023.pdf?sequence=8&isAllowed=y.

[13] Torres-Peña J.D., Garcia-Rios A., Delgado-Casado N., Gomez-Luna P., Alcala-Diaz J.F., Yubero-Serrano E.M., Gomez-Delgado F., Leon-Acuña A., Lopez-Moreno J., Camargo A. (2018). Mediterranean Diet Improves Endothelial Function in Patients with Diabetes and Prediabetes: A Report from the CORDIOPREV Study. Atherosclerosis.

[14] Murie-Fernandez M., Irimia P., Toledo E., Martínez-Vila E., Buil-Cosiales P., Serrano-Martínez M., Ruiz-Gutiérrez V., Ros E., Estruch R., Martínez-González M.Á. (2011). Carotid Intima-Media Thickness Changes with Mediterranean Diet: A Randomized Trial (PREDIMED-Navarra). Atherosclerosis.

[15] Cornejo del Río V., Mostaza J., Lahoz C., Sánchez-Arroyo V., Sabín C., López S., Patrón P., Fernández-García P., Fernández-Puntero B., Vicent D. (2017). Prevalence of Peripheral Artery Disease (PAD) and Factors Associated: An Epidemiological Analysis from the Population-Based Screening PRE-Diabetes and Type 2 DIAbetes (SPREDIA-2) Study. PLoS ONE.

[16] Wang D., Karvonen-Gutierrez C.A., Jackson E.A., Elliott M.R., Appelhans B.M., Barinas-Mitchell E., Bielak L.F., Huang M.-H., Baylin A. (2020). Western Dietary Pattern Derived by Multiple Statistical Methods Is Prospectively Associated with Subclinical Carotid Atherosclerosis in Midlife Women. J. Nutr..

[17] Gardener H., Wright C.B., Cabral D., Scarmeas N., Gu Y., Cheung K., Elkind M.S.V., Sacco R.L., Rundek T. (2014). Mediterranean Diet and Carotid Atherosclerosis in the Northern Manhattan Study. Atherosclerosis.

[18] Lechner K., von Schacky C., McKenzie A.L., Worm N., Nixdorff U., Lechner B., Kränkel N., Halle M., Krauss R.M., Scherr J. (2020). Lifestyle Factors and High-Risk Atherosclerosis: Pathways and Mechanisms beyond Traditional Risk Factors. Eur. J. Prev. Cardiol..

[19] Kumar A., Kar S., Fay W.P. (2011). Thrombosis, Physical Activity, and Acute Coronary Syndromes. J. Appl. Physiol..

[20] Walker T.J., Heredia N.I., Lee M., Laing S.T., Fisher-Hoch S.P., McCormick J.B., Reininger B.M. (2019). The Combined Effect of Physical Activity and Sedentary Behavior on Subclinical Atherosclerosis: A Cross-Sectional Study among Mexican Americans. BMC Public Health.

[21] Jae S.Y., Lee K.H., Kim H.J., Kunutsor S.K., Heffernan K.S., Climie R.E., Bunsawat K., Kang M. (2022). Separate and Joint Associations of Cardiorespiratory Fitness and Healthy Vascular Aging With Subclinical Atherosclerosis in Men. Hypertension.

[22] van de Laar R.J., Ferreira I., van Mechelen W., Prins M.H., Twisk J.W., Stehouwer C.D. (2010). Lifetime Vigorous But Not Light-To-Moderate Habitual Physical Activity Impacts Favorably on Carotid Stiffness in Young Adults. Hypertension.

[23] Germano-Soares A.H., Andrade-Lima A., Menêses A.L., Correia M.A., Parmenter B.J., Tassitano R.M., Cucato G.G., Ritti-Dias R.M. (2018). Association of Time Spent in Physical Activities and Sedentary Behaviors with Carotid-Femoral Pulse Wave Velocity: A Systematic Review and Meta-Analysis. Atherosclerosis.

[24] Spring B., Moller A.C., Colangelo L.A., Siddique J., Roehrig M., Daviglus M.L., Polak J.F., Reis J.P., Sidney S., Liu K. (2014). Healthy Lifestyle Change and Subclinical Atherosclerosis in Young Adults: Coronary Artery Risk Development in Young Adults (CARDIA) Study. Circulation.

[25] Fang X., Zhang X., Yang Z., Yu L., Lin K., Chen T., Zhong W. (2024). Healthy Lifestyles and Rapid Progression of Carotid Plaque in Population with Atherosclerosis: A Prospective Cohort Study in China. Prev. Med. Rep..

[26] Wang D., Jackson E.A., Karvonen-Gutierrez C.A., Elliot M.R., Harlow S.D., Hood M.M., Derby C.A., Sternfeld B., Janssen I., Crawford S.L. (2019). Healthy Lifestyle During the Midlife Is Prospectively Associated With Less Subclinical Carotid Atherosclerosis: The Study of Women’s Health Across the Nation. J. Am. Heart Assoc..

[27] Tedla Y.G., Gepner A., Stein J.H., Delaney J.A., Liu C., Greenland P. (2022). Optimal Lifestyle Behaviors and 10-year Progression of Arterial Stiffness: The Multi-Ethnic Study of Atherosclerosis. J. Clin. Hypertens..

[28] Rojo-López M.I., Bermúdez-López M., Castro E., Farràs C., Torres G., Pamplona R., Lecube A., Valdivielso J.M., Fernández E., Julve J. (2023). Low Adherence to the Mediterranean Diet Is Associated with Increased Prevalence and Number of Atherosclerotic Plaques in the ILERVAS Cohort. Atherosclerosis.

[29] Betriu À., Farràs C., Abajo M., Martinez-Alonso M., Arroyo D., Barbé F., Buti M., Lecube A., Portero M., Purroy F. (2016). Randomised Intervention Study to Assess the Prevalence of Subclinical Vascular Disease and Hidden Kidney Disease and Its Impact on Morbidity and Mortality: The ILERVAS Project. Nefrologia.

[30] Sánchez E., Betriu À., López-Cano C., Hernández M., Fernández E., Purroy F., Bermúdez-López M., Farràs-Sallés C., Barril S., Pamplona R. (2019). Characteristics of Atheromatosis in the Prediabetes Stage: A Cross-Sectional Investigation of the ILERVAS Project. Cardiovasc. Diabetol..

[31] Schröder H., Fitó M., Estruch R., Martínez-González M.A., Corella D., Salas-Salvadó J., Lamuela-Raventós R., Ros E., Salaverría I., Fiol M. (2011). A Short Screener Is Valid for Assessing Mediterranean Diet Adherence among Older Spanish Men and Women. J. Nutr..

[32] Sánchez M., Sánchez E., Hernández M., González J., Purroy F., Rius F., Pamplona R., Farràs-Sallés C., Gutiérrez-Carrasquilla L., Fernández E. (2019). Dissimilar Impact of a Mediterranean Diet and Physical Activity on Anthropometric Indices: A Cross-Sectional Study from the ILERVAS Project. Nutrients.

[33] Estruch R., Martínez-González M.A., Corella D., Salas-Salvadó J., Ruiz-Gutiérrez V., Covas M.I., Fiol M., Gómez-Gracia E., López-Sabater M.C., Vinyoles E. (2006). Effects of a Mediterranean-Style Diet on Cardiovascular Risk Factors: A Randomized Trial. Ann. Intern. Med..

[34] Craig C.L., Marshall A.L., Sjöström M., Bauman A.E., Booth M.L., Ainsworth B.E., Pratt M., Ekelund U., Yngve A., Sallis J.F. (2003). International Physical Activity Questionnaire: 12-Country Reliability and Validity. Med. Sci. Sport. Exerc..

[35] Sánchez E., Betriu À., Salas-Salvadó J., Pamplona R., Barbé F., Purroy F., Farràs C., Fernández E., López-Cano C., Mizab C. (2020). Mediterranean Diet, Physical Activity and Subcutaneous Advanced Glycation End-Products’ Accumulation: A Cross-Sectional Analysis in the ILERVAS Project. Eur. J. Nutr..

[36] Grundy S.M., Cleeman J.I., Daniels S.R., Donato K.A., Eckel R.H., Franklin B.A., Gordon D.J., Krauss R.M., Savage P.J., Smith S.C.J. (2005). Diagnosis and Management of the Metabolic Syndrome: An American Heart Association/National Heart, Lung, and Blood Institute Scientific Statement. Circulation.

[37] World Health Organization (1999). Obesity: Preventing and Managing the Global Epidemic.

[38] Marrugat J., D’Agostino R., Sullivan L., Elosua R., Wilson P., Ordovas J., Solanas P., Cordón F., Ramos R., Sala J. (2003). An Adaptation of the Framingham Coronary Heart Disease Risk Function to European Mediterranean Areas. J. Epidemiol. Community Health.

[39] Stein J.H., Korcarz C.E., Hurst R.T., Lonn E., Kendall C.B., Mohler E.R., Najjar S.S., Rembold C.M., Post W.S. (2008). Use of Carotid Ultrasound to Identify Subclinical Vascular Disease and Evaluate Cardiovascular Disease Risk: A Consensus Statement from the American Society of Echocardiography Carotid Intima-Media Thickness Task Force. Endorsed by the Society for Vascul. J. Am. Soc. Echocardiogr..

[40] Touboul P.-J., Hennerici M.G., Meairs S., Adams H., Amarenco P., Desvarieux M., Ebrahim S., Fatar M., Hernandez Hernandez R., Kownator S. (2004). Mannheim Intima-Media Thickness Consensus. Cerebrovasc. Dis..

[41] Martínez-González M.A., García-Arellano A., Toledo E., Salas-Salvadó J., Buil-Cosiales P., Corella D., Covas M.I., Schröder H., Arós F., Gómez-Gracia E. (2012). A 14-Item Mediterranean Diet Assessment Tool and Obesity Indexes among High-Risk Subjects: The PREDIMED Trial. PLoS ONE.

[42] StataCorp (2019). Stata Statistical Software: Release 16.

[43] R Core Team (2018). R: A Language and Environment for Statistical Computing.

[44] Sala-Vila A., Romero-Mamani E.-S., Gilabert R., Núñez I., de la Torre R., Corella D., Ruiz-Gutiérrez V., López-Sabater M.-C., Pintó X., Rekondo J. (2014). Changes in Ultrasound-Assessed Carotid Intima-Media Thickness and Plaque With a Mediterranean Diet. Arter. Thromb. Vasc. Biol..

[45] Ibanez B., Fernández-Ortiz A., Fernández-Friera L., García-Lunar I., Andrés V., Fuster V. (2021). Progression of Early Subclinical Atherosclerosis (PESA) Study. J. Am. Coll. Cardiol..

[46] Mateo-Gallego R., Uzhova I., Moreno-Franco B., León-Latre M., Casasnovas J.A., Laclaustra M., Peñalvo J.L., Civeira F. (2017). Adherence to a Mediterranean Diet Is Associated with the Presence and Extension of Atherosclerotic Plaques in Middle-Aged Asymptomatic Adults: The Aragon Workers’ Health Study. J. Clin. Lipidol..

[47] Casas R., Urpi-Sardà M., Sacanella E., Arranz S., Corella D., Castañer O., Lamuela-Raventós R.-M., Salas-Salvadó J., Lapetra J., Portillo M.P. (2017). Anti-Inflammatory Effects of the Mediterranean Diet in the Early and Late Stages of Atheroma Plaque Development. Mediat. Inflamm..

[48] IPAQ Research Committee Guidelines for Data Processing and Analysis of the International Physical Activity Questionnaire; IPAQ: 2005. https://sites.google.com/view/ipaq/score.

[49] Aengevaeren V.L., Mosterd A., Braber T.L., Prakken N.H.J., Doevendans P.A., Grobbee D.E., Thompson P.D., Eijsvogels T.M.H., Velthuis B.K. (2017). Relationship Between Lifelong Exercise Volume and Coronary Atherosclerosis in Athletes. Circulation.

[50] Madssen E., Moholdt T., Videm V., Wisløff U., Hegbom K., Wiseth R. (2014). Coronary Atheroma Regression and Plaque Characteristics Assessed by Grayscale and Radiofrequency Intravascular Ultrasound After Aerobic Exercise. Am. J. Cardiol..

[51] Nishitani-Yokoyama M., Miyauchi K., Shimada K., Yokoyama T., Ouchi S., Aikawa T., Kunimoto M., Yamada M., Honzawa A., Okazaki S. (2018). Impact of Physical Activity on Coronary Plaque Volume and Components in Acute Coronary Syndrome Patients After Early Phase II Cardiac Rehabilitation. Circ. J..

[52] Merghani A., Maestrini V., Rosmini S., Cox A.T., Dhutia H., Bastiaenan R., David S., Yeo T.J., Narain R., Malhotra A. (2017). Prevalence of Subclinical Coronary Artery Disease in Masters Endurance Athletes with a Low Atherosclerotic Risk Profile. Circulation.

[53] Aengevaeren V.L., Mosterd A., Bakker E.A., Braber T.L., Nathoe H.M., Sharma S., Thompson P.D., Velthuis B.K., Eijsvogels T.M.H. (2023). Exercise Volume Versus Intensity and the Progression of Coronary Atherosclerosis in Middle-Aged and Older Athletes: Findings From the MARC-2 Study. Circulation.

[54] Chen H., Chen C., Spanos M., Li G., Lu R., Bei Y., Xiao J. (2022). Exercise Training Maintains Cardiovascular Health: Signaling Pathways Involved and Potential Therapeutics. Signal Transduct. Target. Ther..

[55] Tuomilehto J. (2004). Impact of Age on Cardiovascular Risk: Implications for Cardiovascular Disease Management. Atheroscler. Suppl..

[56] Jousilahti P., Vartiainen E., Tuomilehto J., Puska P. (1999). Sex, Age, Cardiovascular Risk Factors, and Coronary Heart Disease. Circulation.

[57] Rodgers J.L., Jones J., Bolleddu S.I., Vanthenapalli S., Rodgers L.E., Shah K., Karia K., Panguluri S.K. (2019). Cardiovascular Risks Associated with Gender and Aging. J. Cardiovasc. Dev. Dis..

[58] Man J.J., Beckman J.A., Jaffe I.Z. (2020). Sex as a Biological Variable in Atherosclerosis. Circ. Res..

[59] Bergström G., Persson M., Adiels M., Björnson E., Bonander C., Ahlström H., Alfredsson J., Angerås O., Berglund G., Blomberg A. (2021). Prevalence of Subclinical Coronary Artery Atherosclerosis in the General Population. Circulation.

