# Supplementary material for: Mediterranean Diet Is a Predictor of Progression of Subclinical Atherosclerosis in a Mediterranean Population: The ILERVAS Prospective Cohort Study
Source: Nutrients. 2024 Oct 24;16(21):3607. doi: 10.3390/nu16213607 (PMC11547874; doi:10.3390/nu16213607)
Supplement: Supplementary file 1 [file nutrients-16-03607-s001.zip › nutrients-3243022-supplementary.pdf]

Mediterranean diet is a predictor of progression of subclinical atherosclerosis in a Mediterranean population: the ILERVAS prospective cohort study

Supplementary Materials

Supplementary Figure S1. Flow chart of the study population.

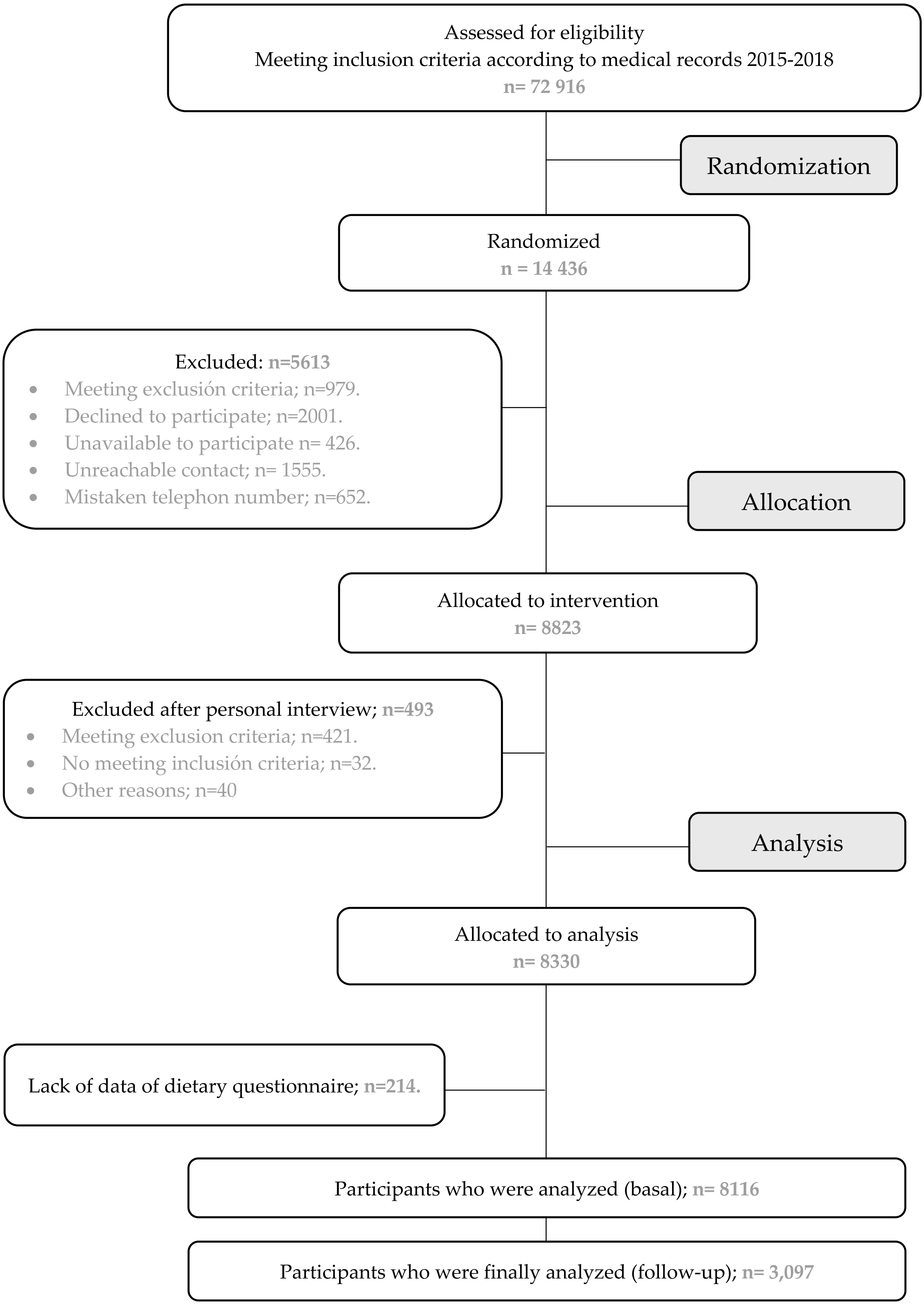

Supplementary Table S1. Multivariable logistic model for the analysis between the Mediterranean diet and physical activity at baseline and progression of plaque

Variables	Plaque progression	
	OR (95% CI)	p
**MEDAS Score**	0.98 (0.93-1.02)	0.313
**Physical activity (METS)**	1.00 (1.00-1.00)	0.342
**Age (years)**	1.04 (1.02-1.05)	<0.001
**Sex, women**	0.79 (0.66-0.94)	0.009
**Hypertension**	1.26 (1.06-1.49)	0.007
**Dyslipidemia**	1.35 (1.15-1.60)	<0.001
**Smoking**	1.37 (1.16-1.62)	<0.001
**Higher waist risk (cm)**	0.83 (0.70-1.00)	0.043
**eGFR (mL/min/1.73m^2)**	1.00 (1.00-1.00)	0.697
**Cons**	0.27 (0.09-0.80)	0.020

CI, confidence interval; eGFR, estimated glomerular filtration rate; MEDAS, Mediterranean Diet Adherence Screener; METS, Metabolic Equivalent Tasks; OR, odds ratio; p, p-value.
Cons estimates baseline incidence rate.
Note: This multivariable logistic model examines the relationship between adherence to the MDiet (as measured by the MEDAS score), physical activity (measured in METS) separately, and the progression of atherosclerotic plaque.

Supplementary Table S2. Model of linear regression analysis of the number of plaque territories and the summarized score between the baseline and the follow-up

Variables	Estimate	Standard error	p
**Summarized lifestyle score**	0.03	0.03	0.323
**Age (years)**	0.03	0.00	<0.001
**Sex, women**	-0.32	0.05	<0.001
**Hypertension**	0.18	0.05	0.001
**Waist circumference (cm)**	-0.00	0.00	0.042
**Smoking**	0.26	0.05	<0.001
**Dyslipidemia**	0.15	0.05	0.002
**GFR (mL/min/1.73m^2)**	0.00	0.00	0.465
**Intercept**	-1.20	0.50	0.632

eGFR, estimated glomerular filtration rate; p, p-value.
Note: This linear regression model examines the association between the number of plaque territories and their summarized score and various factors between baseline and follow-up. The summarized lifestyle score represents the overall healthiness of lifestyle habits (MDiet and PA).

Supplementary Table S3. Multivariable Poisson model for the analysis between the Mediterranean diet, physical activity and the interaction of smoking and age at baseline and number of territories with plaque at follow-up

Variables	IRR (95% CI)	p
**MEDAS Score**	0.97 (0.96-0.99)	<0.001
**Physical activity (METS)**	1.00 (1.00-1.00)	0.218
**Age (y)**	1.05 (1.04-1.06)	<0.001
**Sex, women**	0.67 (0.63-0.72)	<0.001
**Hypertension**	1.19 (1.13-1.26)	<0.001
**Dyslipidemia**	1.16 (1.10-1.22)	<0.001
**Smoking**	2.77 (1.64-4.66)	<0.001
**Waist risk (cm)**	0.95 (0.89-1.00)	0.050
**eGFR (mL/min/1.73m^2)**	1.00 (1.00-1.00)	0.038
**Interaction Smoking-age**	0.99 (0.98-1.00)	0.014
**Cons**	0.16 (0.10-0.27)	<0.001

CI, confidence interval; eGFR, estimated glomerular filtration rate; IRR, incidence rate ratio; MEDAS, Mediterranean Diet Adherence Screener; METS, Metabolic Equivalent Tasks; p, p-value.
Cons estimates baseline incidence rate.
Note: This multivariable Poisson regression model assesses the relationship between adherence to the MDiet (measured by the MEDAS score), PA (measured in METS), separately and the number of territories with plaque at follow-up and the interaction of smoking and age.

Supplementary Table S4. Multivariable Poisson model for the analysis between Lifestyle at baseline and number of territories with plaque at follow-up

Variables	IRR (95% CI)	p
**Lifestyle**		
**Mildly healthy lifestyle**	0.97 (0.90-1.03)	0.305
**Moderately healthy lifestyle**	1.02 (0.95-1.09)	0.599
**Hightly healthy lifestyle**	0.99 (0.92-1.07)	0.775
**Age (years)**	1.04 (1.04-1.05)	<0.001
**Sex, women**	0.67 (0.63-0.71)	<0.001
**Hypertension**	1.19 (1.12-1.25)	<0.001
**Dyslipidemia**	1.16 (1.10-1.22)	<0.001
**Smoking**	1.45 (1.36-1.53)	<0.001
**Waist risk (cm)**	0.95 (0.90-1.00)	0.057
**eGFR (mL/min/1.73m^2)**	1.00 (1.00-1.00)	0.040
**Cons**	0.21 (0.15-0.31)	<0.001

Unhealthy lifestyle was the reference group.
CI, confidence interval; eGFR, estimated glomerular filtration rate; IRR, incidence rate ratio; p, p-value.
Cons estimates baseline incidence rate.
Note: This multivariable Poisson regression model evaluates the relationship between different lifestyle categories at baseline (mildly, moderately, and highly healthy) and the number of territories with plaque at follow-up.
